# Evaluating drug resistance in visceral leishmaniasis: the challenges

**DOI:** 10.1017/S0031182016002031

**Published:** 2016-11-21

**Authors:** S HENDRICKX, PJ GUERIN, G CALJON, SL CROFT, L MAES

**Affiliations:** 1 Laboratory for Microbiology, Parasitology and Hygiene (LMPH), University of Antwerp, Antwerp, Belgium; 2 Nuffield Department of Clinical Medicine, Centre for Tropical Medicine and Global Health, University of Oxford, Oxford, UK; 3 Infectious Diseases Data Observatory, University of Oxford, Oxford, UK; 4 London School of Hygiene & Tropical Medicine, Faculty of Infectious and Tropical Diseases, London, UK

**Keywords:** Visceral leishmaniasis, drug susceptibility, assay procedures, harmonization

## Abstract

For decades antimonials were the drugs of choice for the treatment of visceral leishmaniasis (VL), but the recent emergence of resistance has made them redundant as first-line therapy in the endemic VL region in the Indian subcontinent. The application of other drugs has been limited due to adverse effects, perceived high cost, need for parenteral administration and increasing rate of treatment failures. Liposomal amphotericin B (AmB) and miltefosine (MIL) have been positioned as the effective first-line treatments; however, the number of monotherapy MIL-failures has increased after a decade of use. Since no validated molecular resistance markers are yet available, monitoring and surveillance of changes in drug sensitivity and resistance still depends on standard phenotypic *in vitro* promastigote or amastigote susceptibility assays. Clinical isolates displaying defined MIL- or AmB-resistance are still fairly scarce and fundamental and applied research on resistance mechanisms and dynamics remains largely dependent on laboratory-generated drug resistant strains. This review addresses the various challenges associated with drug susceptibility and -resistance monitoring in VL, with particular emphasis on the choice of strains, susceptibility model selection and standardization of procedures with specific read-out parameters and well-defined threshold criteria. The latter are essential to support surveillance systems and safeguard the limited number of currently available antileishmanial drugs.

## INTRODUCTION: THE GROWING NEED TO DEFINE RESISTANCE

Based on mortality and morbidity, leishmaniasis is currently still one of the world's most neglected tropical infectious diseases (Houweling *et al.* 2016) with the visceral form (VL – visceral leishmaniasis) causing approximately 0·2–0·4 million cases and up to 30 000 deaths worldwide annually (Alvar *et al.* 2012). However, the actual number of VL infected individuals is probably higher due to underreporting and delayed diagnosis (Gurunath *et al.* 2014). The Indian subcontinent alone was responsible for over 60% of the global VL disease burden with almost 50% of all VL cases in Bihar state (India), making it the VL ‘hotspot’ (Bhunia *et al.* 2013; Muniaraj, 2014). Although VL mainly strikes the populations in poverty in developing countries, over the years it has become an emerging problem due to a rise in migration patterns, a lack of control measures and the growing number of HIV/VL coinfections for instance (Ready, 2014). Currently, treatment of human VL relies on a limited number of drugs all with issues that limit their widespread use. While VL was mainly treated with pentavalent antimony (Sb^V^) formulations in the past (Chakravarty & Sundar, 2010; Haldar *et al.* 2011), their first-line use in the Indian subcontinent was largely abandoned due to the emergence of widespread Sb^V^-resistance (Muniaraj, 2014). Although liposomal amphotericin B (AmB) (AmBisome^®^) is currently recommended in the Indian subcontinent within a large-scale VL elimination effort, until recently its use was restricted due its high cost, limited availability and the requirement for cold chain facilities. At present, a large effort is being made to make AmBisome^®^ available at large-scale in endemic areas. In 2011, Gilead partnered with the World Health Organization (WHO) resulting in a large batch of AmBisome^®^ that is now available at discounted prices in developing countries. Following the 2012 London Declaration (Balasegaram *et al*. 2012), it has even been donated by Gilead via the WHO, currently making it the drug of choice for the VL elimination programme in the Indian subcontinent. In addition to its enhanced availability, the KalaCORE programme (www.kalacore.org) aims to improve access and supports cold-chains in India.

In the recent past, miltefosine (MIL) monotherapy was proposed as an effective and more affordable alternative, while the use of paromomycin (PMM) is limited to combinations with Sb^V^ in East Africa (Davidson *et al.* 2009; Thakur *et al.* 2000) or in combination with MIL as a proposed second-line treatment in the Indian subcontinent (Sundar *et al*, 2011). However, several *in vitro* and *in vivo* laboratory studies indicated that development of resistance against both drugs could be expected, even when used in combination therapy (Seifert *et al.* 2003; Garcia-Hernandez *et al.* 2012; Hendrickx *et al.* 2012, 2014). MIL-monotherapy was implemented until 2014 as the first-line option in the kala-azar elimination programme (Jha *et al.* 2013). High relapse rates combined with the possible limited drug exposure in some patients (Rijal *et al.* 2013; Dorlo *et al.* 2014) ultimately led to a revision of this current VL treatment regimen. Although no definite link with intrinsic drug resistance could be established (Rijal *et al.* 2013; Carnielli *et al.* 2014; Hendrickx *et al.* 2015*a*), the use of MIL-monotherapy is now discouraged.

In addition to challenges with drug resistance in human VL, the situation is generally comparable for canine leishmaniasis (canL). While the use of the drugs that are also used to treat human VL is discouraged for canL treatment, there has been widespread use of MIL in veterinary practice. This deserves particular attention as it creates a significant additional selection drug pressure for *Leishmania infantum* (Noli & Saridomichelakis, 2014). Since no cure can be obtained, repeated treatments of infected dogs could select for resistant parasites and enhanced zoonotic transmission to man.

It is essential that in-depth research into the characterization of the factors affecting treatment efficacy and in particular identifying drug resistance mechanisms should acquire more momentum to provide molecular markers for ongoing resistance surveillance which, in turn, will give guidance to physicians and the health community to draw up adapted treatment policies. There is an urgent need to characterize and distinguish between parasite- and host-related effects on drug efficacy. Since a lot of variation in drug susceptibility has already been described between strains from different geographical regions (Hailu *et al.* 2010; Machado *et al.* 2010; Chrusciak-Talhari *et al.* 2011; Soto *et al.* 2004, 2008) a reliable and reproducible method is needed to discriminate shifts in drug susceptibility and actual drug resistance within parasite populations from the host pharmacokinetic and immunological factors. Even though an intensive search for easy-to-use molecular markers has been pursued during the last decade, we still do not have standard criteria and methodologies to unequivocally define a parasite's drug susceptibility. The pivotal role of the patient (immunity, genetic background, etc.) in post-treatment relapses should not be overlooked either.

## DRUG RESISTANCE MARKERS

When talking about drug resistance, it is important to distinguish between drug susceptibility, drug sensitivity and drug resistance. Drug susceptibility is defined as the response of a certain *Leishmania* strain/isolate to a standard drug under defined *in vitro* conditions, whereas drug sensitivity implies measuring the response of the strain/isolate to a standard drug *in vivo* using predefined doses, dose-schedules, and including pharmacokinetics and immune status of the host. Ineffectiveness of killing organisms by what is considered a state-of-the-art treatment generally refers to drug resistance. Since drug susceptibility testing is quite labour-intensive, expensive and time-consuming, access to validated resistance markers that are easier to use in routine laboratory settings would be highly desirable (Croft *et al.* 2006; t'Kindt *et al.* 2010). In this respect, numerous proteomic and metabolomic studies on large sample sets of laboratory and clinical isolates currently aim to identify putative resistance markers and novel drug targets (Scheltema *et al.* 2010; Downing *et al.* 2011; Vanaerschot *et al.* 2012*b*; Berg *et al.* 2013; De Jesus *et al.* 2014). However, the results of such studies are difficult to interpret given their dependence on the parasite stage and culture conditions, and the general pleiotropic resistance mechanisms expected (Silva *et al.* 2011). Molecular characterization is further hampered by the highly plastic nature of the *Leishmania* genome, as reflected by its ability to swiftly modulate gene expression by gene amplification/deletion and to alter its chromosome ploidy in reaction to stress (Ubeda *et al.* 2008; Leprohon *et al.* 2009; Brotherton *et al.* 2013). Given the availability of Sb^V^-resistant clinical isolates, numerous studies have focused on identifying molecular markers responsible for changes in Sb-susceptibility, revealing as the most reported targets aquaglyceroporin 1 (AQP1) (Mandal *et al.* 2010; Kumar *et al.* 2012) and the ATP-binding cassette (ABC) transporter MRPA (Singh, 2006; Ashutosh *et al.* 2007; Mittal *et al.* 2007; Mukherjee *et al.* 2007; Kumar *et al.* 2012), which play a role in drug uptake and sequestration. Other targets suggested to be involved in Sb-resistance are phosphoglycerate kinase (PGK) implicated in glycolysis metabolism (Kazemi-Rad *et al.* 2013), the multidrug-resistance protein 1 (Mukherjee *et al.* 2013), the kinetoplastid membrane protein (El *et al.* 2009), heat-shock protein 83 (HSP83) (Vergnes *et al.* 2007), histone H2A (Singh *et al.* 2010), γ-glutamylcysteine synthase (Ashutosh *et al.* 2012), ornithine decarboxylase (Mukherjee *et al.* 2007), mitogen-activated protein kinase (Ashutosh *et al.* 2012; Kazemi-Rad *et al.* 2013) and protein tyrosine phosphatase (Kazemi-Rad *et al.* 2013). Since variations in gene expression do occur between different strains and species, recent studies recommend expression analysis of multiple genes as a valid biomarker to differentiate Sb-resistance in clinical isolates (Kumar *et al.* 2012; Imamura *et al.* 2016).

For MIL, several studies suggested genetic adaptations in the *Leishmania donovani* putative miltefosine transporter (LdMT) and its cofactor LdRos3 (Perez-Victoria *et al.* 2003*a*, *b*; Perez-Victoria *et al.* 2006*a*; Seifert *et al.* 2007). However, identification of fully reliable molecular markers is still partly hampered by the lack of *in vivo* defined drug-resistant clinical isolates (Rijal *et al.* 2013; Mondelaers *et al.* 2016). Although reports on clinical resistance have appeared for the other two used drugs PMM and AmB (Purkait *et al.* 2012; Hendrickx *et al.* 2014), generally the number of clinical failure isolates associated to drug resistance is still scarce, hence most of our current knowledge on drug resistance has been gathered using laboratory-derived resistant and susceptible parasites. In addition, most laboratory studies have focused on resistance-selected promastigotes rather than the relevant intracellular amastigote stage, which may conceal the real predictive value of the findings.

## DRUG SUSCEPTIBILITY TESTING

### Diagnosis

**Fig. 1.**
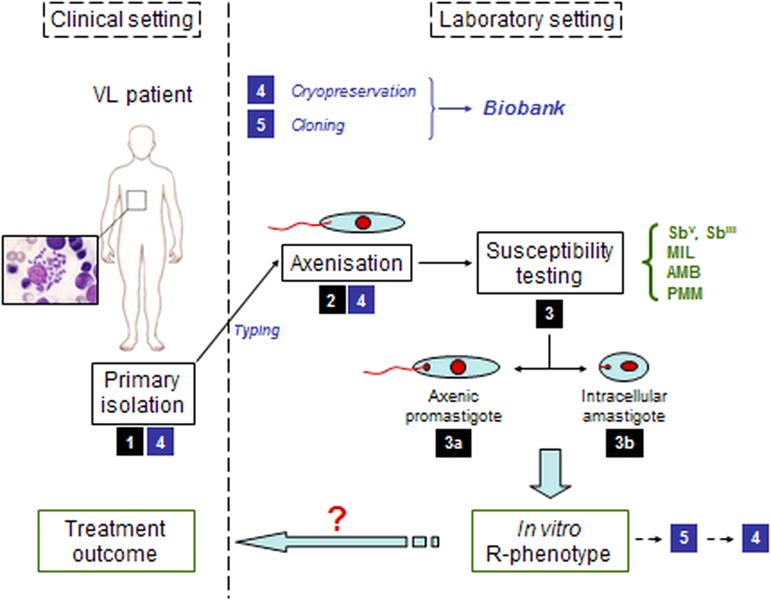
Schematic overview of the pathway from the clinical setting with infected patient to the *in vitro* susceptibility testing of the clinical field isolate in the laboratory setting. (1) primary isolation from infected patient; (2) adaptation of the parasite to *in vitro* culture; (3) susceptibility testing either on a/ promastigotes or b/ intracellular amastigotes; (4) cryopreservation; (5) cloning.

Parasitological diagnosis in the patient still relies on the microscopic examination of bone-marrow or spleen aspirates accompanied by the establishment of *in vitro* aspirate cultures of promastigotes (Sinha *et al.* 1993; Mondal *et al.* 2010; Boelaert *et al.* 2014). Detection of drug resistance still relies on parasite isolation and phenotypic susceptibility testing (Sundar *et al.* 2014). Since these processes are complicated (Fig. 1) and are hampered by safety risks, complexity and slow generation of results (Maes *et al.* 2013; Boelaert *et al.* 2014), parasite isolation for diagnosis has been gradually replaced by non-invasive molecular techniques or immunochromatographic methods detecting *Leishmania* antigens or antibodies in patient sera (WHO, 2010; Boelaert *et al.* 2014).

### Microbiological testing

Given *Leishmania*’s digenetic life cycle, the parasite's drug susceptibility can be determined either on the promastigote vector stage or the amastigote mammalian stage. Most laboratories still routinely establish drug susceptibility of clinical isolates on the axenically cultured promastigote stage. By exposing promastigotes to serial drug dilution series, parasite viability can be read out quite easily with Alamar blue, MTT or resazurin (Fumarola *et al.* 2004; Maes *et al.* 2013). Although a correlation can be found between promastigote and amastigote susceptibility for some drugs (Kulshrestha *et al.* 2013), the intracellular amastigote model is still considered to be the gold standard in antileishmanial drug susceptibility determination given the stage-dependent drug efficacy (Vermeersch *et al.* 2009). To determine the intracellular amastigote susceptibility, macrophages are infected with either metacyclic promastigotes (routinely available upon parasitological diagnosis) or organ-derived amastigotes (generally after adaptation to laboratory rodents). After infection, serial drug dilutions are added to the infected cells and the amastigote burden reduction compared to untreated control cells is determined microscopically, making this assay time-consuming, labour-intensive and therefore quite expensive. Given the stage-specific difference in drug susceptibility, the use of axenic amastigotes is still under debate as there are differences in drug susceptibility compared with the intracellular amastigote (Gupta *et al.* 2001; Vermeersch *et al.* 2009). Although assays using luminescent, fluorescent or colorimetric assays are useful for drug screening, the genetic manipulation of the isolate and time-in-culture makes this approach redundant in this context. When it comes to designing an assay for intracellular amastigotes, the use of macrophages of diverse origins (e.g. different cell lines *vs* primary macrophages) (Seifert *et al.* 2010), different culture media and the use of different methods of infection and treatment protocols all contribute to the fact that direct comparison of susceptibility data between different laboratories is virtually impossible without agreed standardization of protocols and analysis (Table 1).

**Table 1.** Factors involved in the proposed standardization

1.	Species, strain of *Leishmania*
2.	Methodology
	A. Parasite stage B. Host cell type
3.	Medium
4.	Inoculum
5.	Incubation temperature
6.	Incubation time
7.	Compound/drug formulation
8.	Endpoint
9.	Quality control
	A. Number of replicates/repeats B. Reference drugs and acceptable range C. Background signal ratio

### Defining drug resistance

In the absence of easy ways to assess *in vivo* resistance due to the large number of factors affecting host response, *in vitro* techniques are generally considered for defining drug resistance. It specifically refers to a particular decrease of susceptibility of a certain *Leishmania* strain or species to a standard drug under the same predefined *in vitro* conditions and falsely assumes that the initial susceptibility of the parasite population before treatment is always known (Croft *et al.* 2006). In order to define ‘*in vitro*’ resistance, a drug concentration ‘threshold’ should be selected that is able to distinguish a susceptible population from a non-susceptible one. Concentration thresholds or putative molecular markers can only be defined after extensive susceptibility studies on hundreds or thousands of clinical isolates over varying regions, as has been established for malaria by the WorldWide Antimalarial Resistance Network (WWARN) (www.wwarn.org) and for drug resistant bacteria in Europe by the European Committee on Antimicrobial Susceptibility Testing (EUCAST). Tentatively proposed ‘threshold’ values of the current antileishmanial reference drugs Sb^V^, AmB, MIL and PMM for all species of the *L. donovani* complex are summarized in Table 2 (Maes *et al.* 2013). As an alternative, the use of an activity or resistance index has been suggested, expressing the fold decrease of susceptibility of a certain isolate compared with a drug susceptible reference isolate (Yardley *et al.* 2006; Inocencio da Luz *et al.* 2009). For some drugs, this approach is severely hampered by the natural variations in susceptibility of drug-responsive clinical isolates, precluding definition of clear cut-off values for resistance.

**Table 2.** ‘Breakpoint’ estimates^a^ for categorizing drug-susceptibility and drug resistance against antimonials (Sb), miltefosine (MIL), paromomycin (PMM) and amphotericin B (AmB)

	Promastigotes (axenic)	Amastigotes (primary mouse macrophages)	Susceptibility limits (IC_50_ estimates)	Cytotoxicity (MRC-5)	
Drug^b^	IC_50_	IC_50_	sensitive	resistant	CC_50_
Sb^V^	>77	10–15	<20	>70	>64
Sb^III^	40–50	5–6	<15	>70	>64
MIL	2–5	3–6	<10	>25^c^	32
PMM	15–25	40–50	<60	>150^c^	>500
AmB	0·1–0·3	0·01–0·03	<0·5	(>2)^c^	>8

a Based on results obtained with sensitive reference strains (*L. donovani* MHOM/ET/67/L82 and *L. infantum* MHOM/MA/67/ITMAP263).

b *μ*g mL^−1^ for Sb, *μ*m for other compounds.

c at present, no resistant isolates from treated patients are available yet.

### Hurdles encountered during in vitro susceptibility testing

Defining a resistance ‘threshold’ concentration is further complicated by the known variation in species and strain drug-responsiveness, making the establishment of a single ‘threshold value extremely challenging. Next to these interspecies differences and the genetic diversity linked to geographical background, the polyclonal nature of strains (Fernandez *et al.* 2012; Hendrickx *et al.* 2012) also precludes extension of conclusions on susceptibility/resistance between species and hence enforces similar susceptibility studies for every *Leishmania* species and strain (Gouzelou *et al.* 2013).

Drug susceptibility testing of field isolates is logically assumed to be the most useful method to predict treatment outcome, as has been established for antibiotics in bacterial infections (Boothe, 2010) and for malaria (Duru *et al.* 2015). However, treatment failure is a complex interplay between various factors (Table 3) related to either drug, parasite or host, and is thus not necessarily exclusively linked to drug susceptibility (Vanaerschot *et al.* 2014). For example for Sb^V^, a correlation was found between treatment outcome and the *in vitro* amastigote susceptibility profile of field isolates ranging from susceptible (S/S) over intermediate (R/S) to resistant (R/R). Actually, R/R cases could be linked to non-responders or relapse cases while S/S strains were linked to cure. The intermediate R/S profile was linked to an increased risk for R/R development (Inocencio da Luz *et al.* 2009). On the other hand for MIL, *in vitro* susceptibility data could not be used to predict treatment failure (Hendrickx *et al.* 2015*a*) even though some studies do suggest a weak link between *in vitro* susceptibility and treatment outcome (Bhandari *et al.* 2012; Rijal *et al.* 2013). Also the promastigote back-transformation assay, initially proposed as an alternative to assess treatment outcome (Hendrickx *et al.* 2014) could not differentiate between responder and relapse patients when evaluated on a larger set of clinical isolates (Hendrickx *et al.* 2015*a*).

**Table 3.** Overview of factors involved in VL disease progression and treatment failure

Factor	References
Host	
Immunological factors	(Jarvis & Lockwood, 2013; Ostyn *et al.* 2014)
Pharmacokinetics	(Ostyn *et al.* 2014)
Genetics	(Blackwell, 1996; Castellucci *et al.* 2014)
Reinfection	(Rijal *et al.* 2013)
Environment	(Perry *et al.* 2013)
Drug	
Drug quality	(Dorlo *et al.* 2012*a*, *b*)
Intrinsic drug properties (e.g. T_1/2_)	(Hastings *et al.* 2002)
Treatment duration	(Geli *et al.* 2012)
Non-compliance	(Rijal *et al.* 2013)
Parasite	
Lower intrinsic susceptibility to the drug	(Singh *et al.* 2006; Rijal *et al.* 2013)
– gene amplification of drug target enzymes	
– structural and functional modifications of drug target enzymes	(Mondelaers *et al.* 2016)
– decrease in intracellular drug concentration due to extrusion by specific transporters	
Higher parasite fitness	(Vanaerschot *et al.* 2014; Hendrickx *et al.* 2015*b*; Hendrickx *et al.* 2016)

Probably even more important is the fact that, although several labs worldwide have been involved in susceptibility determination of clinical isolates, drug susceptibility values remain fairly inconclusive due the lack of validated standard operating procedures (SOPs), precluding direct comparison of results between laboratories mainly due to large variation in susceptibility assay protocols and endpoints. Even for susceptibility research within the same laboratory, either for antimicrobials, antiparasitics or antifungals, results may vary significantly in time and between replicate tests (Rex *et al.* 1997). It is therefore essential to improve harmonization of laboratory assays while also recognizing that this may not be straightforward. A panel of SOPs and well-defined procedures for quality control would allow better comparison of results, strengthen statistical analysis and could eventually contribute to establish well-defined endpoints and drug resistance breakpoints. For malaria, for example, a special resistance network was founded (WWARN: WorldWide Antimalarial resistance Network; www.wwarn.org; now operating under the auspices of the newly established Infectious Diseases Data Observatory – IDDO) which focuses on the surveillance of drug efficacy by providing detailed procedures for drug preparation, experimental protocols, a tool to generate *in vitro* IC_50_ and IC_90_ values, and literature reviews thereby ensuring that all information generated on antimalarial drug resistance remains of the highest quality and are searchable in a single place (Lourens *et al.* 2010; Sibley & Price, 2012; Woodrow *et al.* 2013). Furthermore, the development of external quality assurance programmes has provided critical tools to compare results across laboratories (Lourens *et al.* 2010; Lourens *et al.* 2014). Unfortunately, there have been few attempts in leishmaniasis to tackle the innumerable challenges to standardize laboratory procedures from the initial stages of parasite isolation and sample propagation, even though some basic variability can never be avoided (Fig. 1). While the introduction of an activity index provided a useful tool to compare experiments between laboratories and between different experiment series (Yardley *et al.* 2006), implementation of stricter ‘drug susceptibility procedures’ will add to the quality of monitoring programmes in the field and even facilitate discovery research for novel antileishmanial drugs. Despite several calls for standardization in the past (Croft, 2001; Croft *et al.* 2006), endpoint criteria and assays required to obtain qualitative data have so far only been defined for natural products (Cos *et al.* 2006), although the same rules could obviously also be used for small molecules.

### Launching a harmonization initiative

The added value of standardized *Leishmania* research procedures is obvious. The first required step is the creation of task force or working group to bring experts from academia, diagnostic laboratories, the clinic and public health together with those who have pioneered similar programmes in malaria and microbial infections, to establish and disseminate a critical set of procedures and analytical tools that will define acceptable levels of harmonization and uniformity between different laboratories. To start this process, a first proposal of some basic procedures concerning diagnostic sampling and follow-up standard laboratory procedures is presented in the Supplementary Material Section (Table 4). The various efforts by the WWARN platform enabled a better comprehension of the factors affecting drug efficacy which subsequently has been corrected by policy changes. For example, WWARN developed a unique on-line model (http://www.wwarn.org/tools-resources/external-quality-assurance) to facilitate individual patient data sharing and engaged over 260 partners around the world, comprised research institutions, governmental and non-governmental organizations, product development partnerships and pharmaceutical companies. These examples of impact should encourage a prompt response from the *Leishmania* research community and launch a similar action plan in support of the elimination of VL from India, Nepal and Bangladesh by 2017 as public health priority (WHO, 2015).

**Table 4.** Overview of the operating procedures presented in the supplementary material section

Procedure	Title
1	Biphasic culture media for primary isolation of promastigotes from spleen- or bone-marrow aspirates
2	Monophasic culture media for axenisation of promastigotes
3	Cryopreservation of *Leishmania* promastigotes
4	Cloning of *Leishmania* promastigotes
5	Preparation of stock solutions of antileishmania reference drugs
6	Drug-susceptibility assay for promastigotes
7	Drug-susceptibility assay for intracellular amastigotes

Beyond this initial action, there are still some aspects that deserve particular consideration in drug resistance research. Given the paucity of drug-resistant clinical isolates, resistance research for most drugs has mainly relied on laboratory-selected strains or on unmatched clinical isolates, hence drifting away from a comparable genetic and phenotypic background. The lack of paired pre- and post-treatment isolates obscures correct interpretation of shifting drug susceptibility. Additionally, the long adaptation process from initial isolation until evaluation in the laboratory further impairs the acquisition of representative clinical isolates, partly related to a changing virulence and genomic profile (Silva *et al.* 2011; Moreira *et al.* 2012). Although previous research already proposed preconditioning of promastigotes as a way to increase and synchronize infectivity and virulence *in vitro* (Inocencio da Luz *et al.* 2009), its systematic use may be debatable as it will affect the original strain-specific phenotypic characteristics.

As already stated, most research has focused on Sb^V^-resistance as large numbers of Sb-resistant clinical isolates were indeed available in India, making laboratory selection of Sb-resistance uncommon. However, as there are relatively few novel antileishmanial compounds in development (www.dndi.org), greater vigilance is warranted and attention must be paid to proactively address treatment failures and relapses in an attempt to contain the risk of drug resistance. Laboratory selection of resistance strains has long been established as a valid tool to study phenotypic changes accompanied by the selection of drug resistance and to unravel underlying resistance mechanisms. The most important benefit of experimental resistance selection is the availability of matched pairs in which the acquired drug resistance undeniably results from the incurred genomic, proteomic and phenotypic variations. However, the relevance of laboratory selected resistant strains exposed to constant drug concentrations in plastic vessels to the ‘real world’ pharmacokinetic variation of drug concentration and different physiological conditions (e.g. oxygen tension) in different tissues has not been established (MacGowan *et al.* 2001).

Many laboratories still select for drug resistance on the promastigote vector stage by cyclic exposure to serially increasing drug concentrations (Maarouf *et al.* 1998; Seifert *et al.* 2003; Perez-Victoria *et al.* 2006*b*; Bhandari *et al.* 2014). Selection on axenic promastigotes has already proven to result in different resistance mechanisms than the ones observed in the clinical setting (Goyeneche-Patino *et al.* 2008). Although much more complex and laborious, selection of MIL- and PMM-resistance at the intracellular amastigote level has proven successful both *in vitro* and *in vivo* (Hendrickx *et al.* 2012, 2015*c*). While the lack of active amastigote replication during successive *in vitro* treatment cycles could suggest passive selection of less susceptible strains (Hendrickx *et al.* 2015*d*), the protocol-dependent outcome for PMM strongly endorses the need to use intracellular amastigotes in drug resistance research (Hendrickx *et al.* 2014, 2012) or at least reach for proper validation when using promastigotes. While overlooking the potential involvement of sand fly factors in transmission and infectivity (Bates, 2007) and still requiring large-scale validation with actual resistant clinical isolates, the intracellular amastigote laboratory models do offer a more representative and predictive alternative to promastigote-based models. Although MIL-resistance could be generated relatively easily at the promastigote level (Perez-Victoria *et al.* 2003*a*; Seifert *et al.* 2003), it should be mentioned that the amastigote-based protocols resulted in the selection of only one MIL-resistant isolate of *L. infantum* (Hendrickx *et al.* 2015*d*), which might be suggestive for strain/species-specificity.

## POSSIBLE EPIDEMIOLOGIC IMPLICATIONS OF RESISTANCE

Selection of drug resistance in most organisms generally results in particular disadvantages with regard to successful survival, reproduction and/or transmission between hosts in a given environment, better known as ‘fitness’ (Natera *et al.* 2007; Borrell & Gagneux, 2009; Orr, 2009; Ait-Oudhia *et al.* 2011). For *Leishmania,* fitness is mainly reflected by the parasite's growth potential, infectivity and its ability to be transmitted (Natera *et al.* 2007). The specific impact of resistance remains debatable and likely depends on the particular drug and parasite species (Kink & Chang, 1987; Detke *et al.* 1988; Gazola *et al.* 2001; Al-Mohammed *et al.* 2005). Several studies indicated enhanced infectivity, metacyclogenesis and transmission in Sb-resistant parasites (Vanaerschot *et al.* 2012*a*). Next to the high selection pressure associated with anthroponotic transmission, this increased parasite fitness might partly explain the widespread Sb-resistance in the Indian subcontinent (Ouakad *et al.* 2011; Vanaerschot *et al.* 2011, 2010). As already mentioned, correct interpretation of such studies may be challenging since they compared sample sets of unpaired isolates with different genotypic and phenotypic background traits (Laurent *et al.* 2007). Since *Leishmania* strains are heterogeneous and show genomic plasticity, the use of wild-type (WT) parent and directly derived drug-resistant lines will rule out the involvement of factors other than an altered drug susceptibility phenotype. At present, this has only been achieved with laboratory-selected resistant strains.

For PMM, increased fitness was observed in a *L. donovani* promastigote-selected strain (Bhandari *et al.* 2014) and further corroborated in an *in vitro L. infantum* amastigote-selected clinical isolate (Hendrickx *et al.* 2016). However, in the latter study no obvious fitness benefit could be detected in promastigotes, once again endorsing again the protocol-dependent and possibly species-dependent outcome of selection (Hendrickx *et al.* 2016). The potential fitness benefit on intracellular amastigote replication and the enhanced defence against oxidative and nitrosative stress, combined with the fairly rapid selection of PMM-resistance (Hendrickx *et al.* 2014, 2012), endorse that PMM should only be used in combination therapy. On the other hand for MIL, resistance selection on amastigotes revealed that resistance may arise much slower than originally anticipated (Hendrickx *et al.* 2014) and that contrary to previous findings (Rai *et al.* 2013), the spread of primary MIL-resistant parasites in the field may be hampered by fitness disadvantages (Hendrickx *et al.* 2016). Although the possibility of decreased fitness may seem somewhat reassuring, the increasing number of MIL-treatment failures is nevertheless alarming particularly since the underlying nature of treatment failure still remains largely unidentified. Studies suggest that treatment failures might be linked to MIL's pharmacokinetic properties, poor compliance linked to the long treatment regimen and/or a reduced drug exposure (Rijal *et al.* 2013; Dorlo *et al.* 2014). However, repetitive MIL-treatment in relapse patients would certainly expedite the emergence of primary MIL-resistance. This is particularly worrying for *L. infantum*, which is highly prevalent in HIV co-infected patients and in canL, both conditions which are repeatedly treated with MIL even though complete parasite eradication cannot be achieved, thereby creating a huge parasite reservoir subject to MIL-exposure and resistance selection (Noli & Saridomichelakis, 2014; van Griensven *et al.* 2014). Of particular concern is that the few available MIL-resistant isolates (either clinical isolates or *in vitro* selected on the amastigote level) are indeed all *L. infantum*.

### Concluding remarks

Monitoring and surveillance of drug sensitivity and resistance are essential to safeguard current treatment options and to establish models for the introduction of new drugs in the future. To discriminate resistant and susceptible strains, a clear definition of drug resistance with establishment of resistance ‘breakpoints’ is definitely needed. Since no validated genomic resistance markers are yet available, resistance monitoring will continue to depend on standard *in vitro* parasite susceptibility testing that should focus on the intracellular amastigote whenever possible and better standardized *in vivo* assessment. The current lack of shared procedures should be addressed with priority to allow correct interpretation and comparison of drug susceptibility studies between labs. In addition, monitoring and surveillance of *Leishmania* resistance in the field should definitely include large sample sizes of matched pre-treatment and post-treatment isolates.

Large-scale implementation of particular SOPs will be challenging and require a time and logistic organization. The present paper is a first tentative proposal to launch a harmonization initiative for particular basic and applied *Leishmania* research procedures. Obviously, this proposed set will have to be disseminated and discussed with the whole *Leishmania* research community at international conferences and within network groups (for example, WorldLeish) to acquire input and practical feedback to enable a widely feasible set of recommendations. Through collaborations with the WHO Neglected Tropical Diseases Department (WHO-NTD) and the WHO's Special Programme for Research and Training in Tropical Diseases (WHO-TDR), performance of elimination and control programmes at country level may become enhanced. A special consortium has already been founded by the Bill and Melinda Gates Foundation to ensure suitable surveillance measures to sustain VL elimination In the Indian subcontinent. Joining all these various efforts should eventually pay off and lead to more reliable and comparable laboratory results that will inform the leishmaniasis community worldwide on the efficacy of the current antileishmanial drugs and the emergence and spread of drug resistance.

## Supplementary material

For supplementary material accompanying this paper visit http://dx.doi.org/10.1017/S0031182016002031. click here to view supplementary material
