# Supplementary material for: Evaluating drug resistance in visceral leishmaniasis: the challenges
Source: Parasitology. 2016 Nov 21;145(4):453–63. doi: 10.1017/S0031182016002031 (PMC5989324; doi:10.1017/S0031182016002031)
Supplement: Supplementary file 1 [file S0031182016002031sup001.docx]

**SUPPLEMENTARY MATERIAL**

In the future, it will become increasingly important to standardize all leishmaniasis research to achieve as much harmonization and uniformity as possible. Applying agreed methodologies might allow comparison between different academic and diagnostic clinical research facilities enabling reliable statistical analysis and thus providing sufficient confidence in defining definite efficacy endpoint and breakpoints for drug resistance. Table S1 summarizes all criteria that might require revision and discussion to achieve standardized assays, while Table S2 offers a first incentive to harmonize a set of laboratory procedures that are commonly applied during sampling and laboratory susceptibility testing. Among academics, it might be recommended to form a task force or working group to discuss and agree on the methodologies and systems needed for the implementation of standardized quality-controlled research in the leishmaniasis community. At a later stage, international organizations such as the World Health Organization (WHO) and Drugs for Neglected Diseases initiative (DNDi) should become involved for the implementation in the clinical setting.

Table S1: Criteria that require a clear description to attain standardized assays.

| **Experimental setting** |
| --- |
| Species, pathogen strain |
| Pathogen stage |
| Host cell type for intracellular assay |
| Culture medium |
| Pathogen inoculum (dose) |
| Incubation temperatures and times |
| Drug formulations |
| **Validation** |
| Standard reference strains |
| Standard drugs (source/batch number)  Proficiency programme |
| **Endpoints** |
| Microscopy, fluorescence, etc. |
| Method of counting  Modelling to estimate optimal endpoints |
| **Quality control** |
| Number of replicates/ assays and repeats |
| Background/signal ratio |
| Control drugs and acceptable range |

Table S2: Provisional suggestion for some commonly used procedures involved in *Leishmania* susceptibility testing ^45^.

A more detailed initial proposal is provided in the section below for all procedures indicated with *

| **Standard operating procedures** | |  | | **Comments** | | **References** | |
| --- | --- | --- | --- | --- | --- | --- | --- |
| Sampling |  |  | |  | |  | |
| *1. Isolation of promastigotes from spleen or bone marrow isolates* | | | | | |  | |
|  | Use of biphasic culture media ***** |  | | Biphasic blood-based media are considered the most appropriate for primary parasite isolation | |  | |
|  | A. Biphasic NNN (Novy McNeal Nicolle) medium | | |  |  | **^1, 2^** | |
|  | B. Evan's modified Tobie's medium | | |  |  |  | |
| Laboratory procedures | |  | |  | |  | |
| *2. In vitro cultivation of axenic promastigotes* | | | |  | |  | |
|  | Use of monophasic culture media ***** | | | Transfer to monophasic media is needed to allow the large volume cultivation necessary for susceptibility testing | |  | |
|  | A. (HO)MEM |  | |  |  |  | |
|  | B. M199 |  | |  |  |  | |
| *3. Cryopreservation of promastigotes* ******* | |  | |  | |  | |
|  | Cryoprotectant: 5% DMSO or 10% glycerol | | - Long-term *in vitro* cultivation of axenic promastigotes is contraindicated on account of loss of virulence | | **^1, 3^** | |  |
|  | Long-term storage in liquid nitrogen (-196°C) | |  |  |  | |  |
|  |  |  | - As quickly as possible after primary isolation | |  | |  |
| *4. Cloning of Leishmania promastigotes* ******* | |  | |  | |  | |
|  | Microdrop dilution method |  | | Always use microscopic evaluation by two independent observers to assess the presence of only one promastigote | | **^4^** | |
|  |  |  | |  |  |  | |
| *5. Preparation of antileishmanial drug solutions* ******* | | | |  | |  | |
| Antileishmanial reference drugs | |  | | - Optimal storage conditions must be used to guarantee chemical stability  - All stock solutions can be made in MilliQ  - Avoid commercial drug formulations with vehicle constituents and preservatives | | **^5, 6^** | |
|  | A. Pentavalent antimony (Sb^V^) |  | |  |  |  | |
|  | B. Trivalent antimony (Sb^III^) |  | |  |  |  | |
|  | C. Amphotericin-B (AmB) |  | |  |  |  | |
|  | D. Miltefosine (MIL) |  | |  |  |  | |
|  | E. Paromomycin (PMM) |  | |  |  |  | |
| *6. Promastigote susceptibility testing* ******* | |  | |  | |  | |
|  | Use of resazurin |  | | - Less attractive alternatives are microscopic counting or the use of MTT of XTT | | **^7-9^** | |
|  |  |  | |  |  |  | |
|  | Promastigote |  | | - Use of 3-day old log phase promastigote cultures | |  | |
|  |  |  | | - Drug exposure for 72h at 25°C | |  | |
|  |  |  | | **! Sb^V^ is inactive against the promastigote stage** | | **^10^** | |
| *7. Drug susceptibility assay for intracellular amastigotes* ******* | | | |  | |  | |
|  | Use of primary peritoneal mouse macrophages | | | - existence of cell-dependent variations | | **^11^** | |
|  | Metacyclogenesis of promastigotes | | | - important to include a drug sensitive reference strain and calculate an activity index (AI) | | **^12^** | |
|  | A. Spontaneous acidification of culture medium | | |  |  | **^13^** | |
|  | B. Preconditioning of promastigotes | | | - other macrophage-like cell lines can be used as well (J774 or THP-1 are most widely used) | |  | |
|  | Microscopic determination of intracellular amastigote burden after Giemsa staining | | |  |  |  | |
|  |  |  |  |  | |  | |
| *8. Experimental resistance induction* | |  | |  | |  | |
|  | Use of primary peritoneal mouse macrophages | | |  | | **^4, 14^** | |
|  | Metacyclogenesis of promastigotes | | |  | |  | |
|  | A. Spontaneous acidification of culture medium | | |  |  |  | |
|  | B. Preconditioning of promastigotes | | |  | |  | |
|  | Microscopic determination of intracellular amastigote burden after Giemsa staining | | |  |  |  | |
|  |  |  |  |  | |  | |
|  | Promastigote back transformation at the IC_90_ before repeating the cycle | | |  | |  | |
|  |  |  |  |  | |  | |

### Procedure 1: Biphasic culture media for primary isolation of promastigotes from spleen- or bone-marrow aspirates

**A. Biphasic NNN (Novy McNeal Nicolle) medium**

- Solid phase (for 100 ml)

Bacto Agar (Difco) 2.0 g

sodium chloride (NaCl) 0.6 g

bi-distilled water 90 ml

*Stir-mix and dissolve the ingredients while heating until the agar melts. Autoclave (121°C, 15 min) and allow to cool down to about 45°C before adding 10 ml rabbit blood*. Quickly aliquot into culture tubes in shallow slant position and allow the agar to solidify. Rapidly cool and store in upright position at 4°C. Culture tubes have to be used within 3 weeks of preparation.*

- Liquid overlay phase: is not needed since enough condensation water will form during the cooling process. If needed, a small amount of distilled water can be added.

**B. Evan’s modified Tobie’s medium**

- Solid phase (for 100 ml)

Bacto Agar (Difco) 1.5 g

Bacto-Tryptose (Difco) 1.5 g

sodium chloride (NaCl) 0.4 g

potassium chloride (KCl) 0.04 g

sodium phosphate (Na_2_HPO_4_.12 H_2_O) 0.5 g

bi-distilled water 80 ml

*Stir-mix and dissolve the ingredients while heating until the agar melts. Autoclave (121°C, 15 min) and store at 4°C. For use, melt in boiling water or in a microwave and allow to cool down to about 45°C before adding 20 ml rabbit blood*. Quickly aliquot into culture tubes (1 ml/tube) in shallow slant position and allow the agar to solidify. Rapidly cool and store in upright position at 4°C (to be used within 3 w of preparation)*

- Liquid overlay phase (Locke solution, for 100 ml)

sodium chloride (NaCl) 0.8 g

potassium chloride (KCl) 0.02 g

potassium phosphate (KH_2_PO_4_) 0.03 g

magnesium sulphate (MgSO4. 7 H_2_O) 0.01 g

sodium bicarbonate (NaHCO_3_) 0.1 g

glucose 0.25 g

bi-distilled water 100 ml

*Dissolve the ingredients and adjust the pH at 7.4 with HCl or NaOH. Filter-sterilize through a 0.22 µm membrane filter and store at 4°C. Depending on the use indication, antibiotics can be added: penicillin 200 I.U/m + streptomycin 200 or gentamycin at 200 µg/ml. Just prior to use, add 1 ml Locke solution to the Tobie agar slant.*

* Blood from rabbits is freshly collected using a syringe prefilled with heparin (25 I.U. heparin/ ml blood)

Remark: always perform a sterility check on each production batch of culture medium. Check for the presence of bacteria after 24h incubation at 37°C.

### Prodecure 2: Monophasic culture media for axenisation of promastigotes

**Materials**

- M199 medium, 10x with Hanks’ salts (Sigma M9163)
- MEM medium (*Invitrogen 31095-029*)
- HEPES (*Sigma H3375*)
- Adenine (*Sigma A2786*)
- Hemin (*Sigma H5533)*
- 6-biopterine (*Schricks Labs 11.203*)
- L-glutamine 200 mM 100x (*Invitrogen 25030-024)*
- Sodium bicarbonate 7.5% (*Invitrogen 25080-060*)
- Folic acid (*Sigma F8758*)
- D-glucose (*Invitrogen 49139*)
- D-biotin (*Sigma B4639*)
- Fetal calf serum (inactivated) (*Invitrogen 10270.106*)
- Triethanolamine (*Sigma T1377*)
- HCl 37% (*Merck biochemicals* [*101834*](http://www.merck-chemicals.com/belgium/hydrochloric-acid-fuming-37-percent/MDA_CHEM-101834/p_uuid?WFSimpleSearch_NameOrID=37%25+HCl&BackButtonText=search+results))
- NaOH (*Sigma S8045*)

**Stock solutions** [stored at 4°C unless otherwise mentioned]

- Hemin stock: 37.5 mg hemin + 15 ml 50% triethanolamine in water (store at –4°C)
- Biopterine stock: 50 mg 6-biopterine + 1 ml HCl 1M + 24 ml distilled water (store at -20°C)
- Adenine stock: 200 mg adenine + 2.5 ml 1M NaOH + 17.5 ml MilliQ water
- Folic acid stock: 15 mg folic acid + 200 µl 1M NaOH + 18.5 ml MilliQ water
- D-biotine stock: 10 mg D-biotine + 500 µl 1M NaOH + 19.5 ml MilliQ water

**Composition of different promastigote media**

| **Ingredients** | **M199** | **HOMEM** |
| --- | --- | --- |
| Culture medium | 50ml M199 10x | 50 ml MEM 10x |
| MilliQ water | 450 ml | 450 ml |
| HEPES | 5 g | 5 g |
| Adenine | 13 mg | 20 mg |
| Hemin stock solution | 2 ml | 2 ml |
| 6-biopterine stock solution | 300 µl | / |
| L-glutamine | 10 ml | 10 ml |
| Sodium bicarbonate 7.5% solution | 2.5 ml | 9 ml |
| Folic acid stock solution | / | 2 ml |
| D-glucose | / | 1.5 g |
| D-biotin stock solution | / | 2 ml |
| Fetal calf serum (inactivated) | 50 ml | 50 ml |

### Procedure 3: Cryopreservation of *Leishmania* promastigotes

**Materials**

- promastigote culture in logarithmic growth with a cell density of at least 10^6^/ml
- cryoprotectant: DMSO (Sigma 41644)
- cryoprotectant: glycerol (Sigma G5516)
- Biofreezer -70°C
- 1.8 ml cryopreservation tubes (Nalgene Nunc # 377267); internal screw stop
- freezing box “Mr. Frosty^®^” (Nalgene Nunc # 9400945), filled with 250 ml room temperature isopropanol up to the line mark (isopropanol has to be changed every fifth use or at least once per month).

**Procedure for freezing**

- Add the cryoprotectant to the promastigote culture under gentle stirring to obtain a final concentration of 5% DMSO or 10% glycerol.
- Aliquot 1 ml cell suspension to each cryotube, firmly close the lid and put the tubes into the freezing box. Place the box immediately into a -70°C freezer for 24 hrs, then into the liquid nitrogen tank.

**Procedure for thawing**

- Remove the cryotube from the liquid nitrogen, and immediately transfer into a 37°C water bath. Hold the tube in the surface of the water bath while very gently and continuously swaying (d*o not leave the cryotube unattended during the thawing process).* With a small bit of ice still remaining in the tube, transfer the tube into the biosafety hood, dry off the outside and wipe with 70% ethanol before opening to prevent contamination.
- Transfer the contents of the tube to 5 ml pre-warmed promastigote medium (37°C) in a culture flask or tube.

**Remarks**

- For quality control on each freezing batch, one tube should be used to start a standard *in vitro* culture; good growth should be obtained within 3 to 4 days.
- For long term storage in liquid nitrogen, DMSO is preferred as cryoprotectant. For storage at -70°C, glycerol is preferably used.
- Using the freezing box “Mr. Frosty^®^” approximates the programmed freezing scheme of: 1°C/min from +18°C to +2°C, then 5°C/min from +2°C to -18°C and finally 10°C/min from -18°C to -70°C.

### Procedure 4: Cloning of *Leishmania* promastigotes

**Materials**

- flat-bottom 96-well microplates, plastic pipet micro-tips
- promastigote culture in logarithmic growth phase
- complete M-199 promastigote culture medium
- ‘spent’ medium: prepared from a logarithmic growth culture after removal of the promastigotes by centrifugation and filtration (0.22 µm) of the supernatant. Aliquots are stored at -20°C.

**Cloning method**

- Dilute the stock culture 1/100 in complete M199 medium and further prepare a two-fold dilution series in a 96-well plate (‘donor plate’). Select the well for probing micro-drops that will deliver one organism/drop with a reasonably high probability.
- The micro-drops are deposited in another 96-well plate (‘cloning plate’) (Figure below).
- Place 8µl complete M199 medium to the side of the wells to minimize evaporation during the manipulations.
- Insert the micro-tip into the appropriate well in the donor plate and deposit a micro-drop by tip touch in the middle of the ‘cloning’ 96-well plate. Several wells can be done before microscopic verification for the presence of one promastigote (*it is advised to have a second person double check*)
- In the wells with one promastigote, 100µl of ‘spent’ M199 medium is added.
- Continue the procedure until all the wells of the cloning zone have been processed.
- Finally, add 100µl of ‘complete’ M199 medium to the wells with a single promastigote. Fill the remaining wells with 200µl milliQ water.
- Wrap the ‘cloning’ plate with parafilm to avoid evaporation and incubate at 25°C.
- Transfer the grown clones (after ± 1 w) into larger culture tubes with M199 medium.

**Figure: Cloning of promastigotes using the micro-drop method in 96-well plates**

### Procedure 5: Preparation of stock solutions of antileishmanial reference drugs

**Preparation of stock solutions**

- Sb^V^ (stock solution at 1 mg Sb^V^ eq /ml)

Dissolve 31.9 mg SSG in 10 ml water or PBS while stirring at 37°C for 1 hour until a clear solution is obtained. Divide into small aliquots (1 ml) and keep at -20°C. Frozen aliquots may be kept for 3 months. [1 mg SSG contains 0.313 mg Sb^V^ eq].

- Sb^III^ (stock solution at 1 mg Sb^III^ eq /ml)

Dissolve 27.7 mg Sb^III^-tartrate in 10 ml water or PBS while stirring until a clear solution is obtained. Divide into small aliquots (1 ml) and keep at -20°C. Frozen aliquots may be kept for 3 months. [1 mg Sb^III^-tartrate contains 0.361 mg Sb^III^ eq].

- AmB (20 mM stock solution)

Dissolve 41 mg Fungizone^®^ powder in 1 ml water to prepare the stock solution. Divide into small aliquots (0.2 ml) and keep at -20°C. Frozen aliquots may be kept for 3 months. Further dilutions are made in water, but should be used immediately because of limited stability. [MW = 924.08 - 1g Fungizone^®^ formulation contains 450 mg AmB].

*Note: Amphotericin B from Sigma (A2411) should not be used because of extreme poor solubility*.

- MIL (20 mM stock solution)

Dissolve 81.5 mg MIL in 10 ml water or PBS while stirring until a clear solution is obtained. Divide into small aliquots (1 ml) and keep at 4°C. Aliquots may be kept for 3 months. (MW = 407.57].

- PMM (20 mM stock solution)

Dissolve 142.74 mg PMM-sulfate in 10 ml water or PBS while stirring until a clear solution is obtained. Divide into small aliquots (1 ml) and keep at 4°C. Aliquots may be kept for 3 months. [MW PMM-sulfate = 713.71; PMM base = 615.63 / salt factor 1.16].

### Procedure 6: Drug-susceptibility assay for promastigotes

**1. Preparation of resazurin stock solution**

- PPB (Potassium Phosphate Buffer 0.1M, pH 7.4)**:** dissolve 2.72 g KH_2_PO_4_ (*Merck 1.04873.1000*) and 18.16 g K_2_HPO_4_.3H_2_O (*Sigma P5504*) in 1L distilled water; store the buffer solution in aliquots at 4°C.
- PPB_a_**:** dissolve resazurin sodium salt (Sigma 199303) at 3 mg/ml in PPB (for example 300 mg in 100 ml PPB).
- PPBb_a_**:** Prepare a 30mM stock of potassium-ferricyanide (K_3_Fe(CN)_6_) *(Sigma P8131)* in PPB (for example 987 mg in 100 ml PPB); can be stored at 4°C until use
- PPB_c_**:** Prepare a 30 mM stock of potassium-ferrocyanide (K_4_Fe(CN)_6_) *(Sigma P9387)* in PPB (for example 1266 mg in 100 ml PPB); can be stored at 4°C until use

*Mix equal volumes of PPB_a_, PPB_b_ and PPB_c_ and dilute the obtained stock solution 1/20 in PPB to obtain the resazurin working solution. Filter-sterilize (0.22 µm) and store in the dark at 4°C. It is advised to prepare a new resazurin solution every 2 weeks.*

**2. Micro-dilution susceptibility assay for promastigotes**

Materials

- '96-well' plates (flat-bottom)
- Resazurin working solution
- Spectrophotometer for fluorimetric reading (filter combination 550 nm / 590 nm)
- Promastigote culture in log-phase growth
- Stock solutions of antileishmania reference drugs (Procedure 5)

Promastigote susceptibility assay

- Log-phase promastigotes are harvested and microscopically counted in a counting chamber (= infection inoculum).
- Promastigotes are seeded into the wells at 10^5^ promastigotes/well and exposed to the serially-diluted drugs in a 96-well plate (specific test plate template and dose range to be designed by the investigator – practical example is presented in Figure below).
- During incubation for 72h at 25°C, plates are wrapped in parafilm to avoid evaporation.
- After the stated incubation period, 50µl of resazurin working solution is added to each well and the plates are further incubated for 24h in the dark.
- Fluorescence reading using the filter combination 550-590nm and calculation of the ED_50_ using appropriate software.

**Figure: Practical template for susceptibility profiling of promastigotes against current antileishmania reference drugs (4 replicates/drug).**

*Control: medium inoculated with promastigotes (= 100% growth)*

*Blank: medium control without promastigotes (= 0% growth)*

Remark: Pentavalent antimony (Sb^V^) has been shown to be inactive against promastigotes and is not included. Although the exact mechanism of action of antimonials is not yet known, it is generally accepted that Sb^V^ is in fact a pro-drugs that requires biological reduction to the trivalent form (Sb^III^). There are indications that Sb^III^ has some activity against the promastigote stage and has for this reason been included in the panel of drugs.

### Procedure 7: Drug-susceptibility assay for intracellular amastigotes

**1. Isolation and culture of primary peritoneal mouse macrophages (MΦ)**

- Complete culture medium: 500 ml RPMI-1640 (Invitrogen 21875-034) supplemented with 10 ml penicillin-streptomycin (Invitrogen 15140-122), 5 ml L-glutamine (Invitrogen 25030-025) and 25 ml heat inactivated fetal bovine serum (FCSi)
- 2% starch-suspension: weigh 0.2 g potato starch into a test tube and semi-sterilize by incubating at 80°C for at least 2h. Add 10ml sterile PBS just before use and stir until all the starch is homogeneously suspended (warm up, if necessary).
- Stimulation and harvesting of macrophages (MΦ): disinfect the abdomen of Swiss mice (± 25 g, male or female) with 70% ethanol and inject 1 ml of the starch suspension intraperitoneally. Macrophages are collected 2 days later after killing the mice with a CO_2_ overdose or cervical dislocation. All next steps are executed in a LAF to maintain sterility. After disinfection of the abdomen with 70% ethanol, 10 ml pre-warmed (37°C) complete RPMI-1640 is injected intraperitoneally, after which the abdomen is gently massaged to distribute the fluid and suspend the cells. After removal of the abdominal skin, the exposed peritoneum is disinfected with 70% ethanol and the abdominal fluid with cells is aspirated. The number of macrophages is determined in a counting chamber and the cell suspension is appropriately diluted with complete RPMI-1640 (37°C) to obtain a cell suspension of about 300,000 macrophages/ml.
- Seeding of macrophages: dispense 100 µl macrophage suspension (30,000 macrophages per well) in each well of a 96-multiwell tissue culture plate and incubate at 37°C and 5% CO_2_ to be used 24 hours later.

**2. Metacyclogenesis of promastigotes ^12^**

Promastigotes in stationary phase are generally used to infect macrophages. However, spontaneous metacyclogenesis of some field isolates is sometimes problematic because of very slow growth. In these cases, pre-conditioning of promastigotes may be useful to enhance cell infectivity and obtain more stable infections.

- Preconditioning of promastigotes: promastigotes are cultured in M199 medium until day 5 (early stationary phase). The promastigotes are separated by centrifugation and the culture medium is replaced by Schneider’s medium at pH 5.4, supplemented with 20% FCSi. The promastigotes are incubated for another 24h at 25°C before being used for infection of macrophages.

**3. *In vitro* susceptibility test in primary mouse macrophages**

- Prepare a suspension of (pre-conditioned) metacyclic promastigotes at 3 x 10^6^/ml in RPMI-1640.
- Add 100 µl promastigote suspension into each well already containing 100 µl macrophage culture. (🡪 infection rate = 10 promastigotes/macrophage) and incubate at 37°C and 5% CO_2_ for 24h.
- Remove the non-internalized promastigotes by discarding the culture medium (200µl) and replenish by adding 190µl RPMI-1640 medium to all the wells, making sure not to touch the monolayer and avoid cross-contamination.
- Finally, transfer with a multichannel pipette 10µl of the reference drug dilutions 20x onto the infected macrophages (avoid touching the monolayer) and incubate in a CO_2_ incubator for 4 days at 37°C.

**4. Determination of intracellular amastigote burdens**

4.1. Giemsa-staining procedure

- 20% Giemsa-solution: prepare a work-solution by diluting Giemsa (Accustain^®^, Sigma) 1/5 in demineralised water and filter (0.22µm membrane filter) to remove particulate matter. Always use freshly prepared staining solution (10 ml work-solution is needed per 96-well plate).
- Staining of microtiter plates: discard the medium from each well of the test plates, allow the plates to dry to the air and dispense 150µl absolute methanol to each well for 10 minutes (fixation). Next, discard the methanol, allow the plates to dry to the air, dispense 100µl Giemsa work-solution in each well and stain for about 15 min. Discard the Giemsa-solution and collect in chemical waste container, rinse the stained plates with tap water and allow drying to the air. The plates are now ready for microscopic reading.

4.2. Determination in intracellular amastigote burdens

- Up till now, microscopic counting is the only option to determine of the total parasite burden, which is calculated as the percentage of infected macrophages X the mean number of amastigotes per macrophage. At least 100 macrophages need to be counted and the infection is judged adequate if at least 70 to 80% of the macrophages are infected.
- The results are expressed as the percent reduction of the total parasite burden compared to the non-treated infected control and the 50% inhibitory concentration (IC_50_) is calculated using appropriate software (for example, Statview^®^)

**Remarks**

- - An almost identical assay template as proposed in Procedure 5 can be used, except that pentavalent antimony (Sb^V^) should now be included (for example, by replacing AmB).
  - For standard drug susceptibility assays, polystyrene microtiter plates have sufficient optical qualities for microscopic evaluation of intracellular amastigote burdens (using a invert microscope with long focus objectives). For more accurate evaluations, Lab-Tek™ culture chambers (Nunc 177445 or 178599) can be used, allowing 500x magnification under oil immersion.
  - In order to more easily compare the results in different experimental series, it is important to include a drug-sensitive reference strain in each test and calculate an activity index (AI), as had been proposed for antimonials.^15^
  - In the case that mice are not available for collection of primary macrophages, other macrophage-like cell types can be used.^11^ Among the different options, J774A.1 and THP-1 cells are most widely used.
  - J774 cells are seeded at 10^5^ cells/well in a 96-well microplate and infected at a multiplicity of infection of 10:1. Plates are microscopically read after Giemsa staining for 10 minutes.
  - THP-1 cells need to be stimulated with phorbol 12-myristate 13-acetate (PMA, Sigma P8139) before becoming adherent. Variable levels of stimulation may influence the test outcome (levels of adherence and infection).

**Figure: Time flow-chart of the intracellular amastigote susceptibility test with indication of the option for pre-conditioning of promastigotes.**

**Figure: Practical template for susceptibility profiling of intracellular amastigotes in MΦ against current antileishmania reference drugs (4 replicates/drug).**

**Reference List**

1.  **Evans, D.A.** (1993) In vitro cultivation and biological cloning of Leishmania. *Methods Molecular Biol*ogy **21**: 29-41.

2.  **Sundar ,S., Rai, M.** (2002) Laboratory diagnosis of visceral leishmaniasis. *Clinical and Diagnostic Laboratory Immunology* **9**: 951-958.

3.  **World Health Organization** (2010) Control of the leishmaniasis: report of a meeting of the WHO Expert Committee on the Control ofLeishmaniases, Geneva, 22-26 March 2010. *WHO technical report series* 2010.

4.  **Hendrickx, S., Inocencio da Luz, R. A., Bhandari, V., Kuypers, K., Shaw, C. D., Lonchamp, J., Salotra, P., Carter, K., Sundar, S., Rijal, S., Dujardin, J. C., Cos, P. and Maes, L.** (2012). Experimental induction of paromomycin resistance in antimony-resistant strains of L. donovani: outcome dependent on in vitro selection protocol. *PLoS Neglected Tropical Diseases,* **6**, e1664. doi: 10.1371/journal.pntd.0001664..

5. **Roberts, W.L., Berman, J.D., Rainey, P.M.** (1995) In vitro antileishmanial properties of tri- and pentavalent antimonial preparations. *Antimicrobial Agents and Chemother*apy, **39**: 1234-1239.

6. **Ephros, M., Waldman, E., Zilberstein, D.** (1997) Pentostam induces resistance to antimony and the preservative chlorocresol in Leishmania donovani promastigotes and axenically grown amastigotes. *Antimicrobial Agents and Chemotherapy,* **41**: 10641068.

7. **Mikus, J., Steverding, D.** (2000) A simple colorimetric method to screen drug cytotoxicity against Leishmania using the dye Alamar Blue. *Parasitoly International,* **48**: 265-269.

8.  **Dutta, A., Bandyopadhyay, S., Mandal, C., Chatterjee, M.** (2005) Development of a modified MTT assay for screening antimonial resistant field isolates of Indian visceral leishmaniasis. *Parasitoly International*, **54**: 119-122.

9. **Williams, C., Espinosa, O.A., Montenegro, H., Cubilla, L., Capson, T.L., Ortega-Barría, E., Romero, L.I.** (2003) Hydrosoluble formazan XTT: its application to natural products drug discovery for Leishmania. *Journal of Microbiological Methods*, **55**: 813-816.

10. **Wyllie, S., Cunningham, M.L., Fairlamb, A.H.** (2004) Dual action of antimonial drugs on thiol redox metabolism in the human pathogen Leishmania donovani. *Journal of Biological Chemistry*, **279**: 39925-39932.

11. **Seifert, K., Escobar, P. and Croft, S. L.** (2010). In vitro activity of anti-leishmanial drugs against Leishmania donovani is host cell dependent. *Journal of Antimicrobial Chemotherapy,* **65**, 508-511. doi: 10.1093/jac/dkp500

12. **Inocencio da Luz, R. A., Vermeersch, M., Dujardin, J. C., Cos, P. and Maes, L.** (2009). In vitro sensitivity testing of Leishmania clinical field isolates: preconditioning of promastigotes enhances infectivity for macrophage host cells. *Antimicrobial Agents and Chemotherapy,* **53**, 5197-5203. doi: 10.1128/AAC.00866-09.

13. **Yardley, V., Ortuno, N., Llanos-Cuentas, A., Chappuis, F., Doncker, S. D., Ramirez, L., Croft, S., Arevalo, J., Adaui, V., Bermudez, H., Decuypere, S. and Dujardin, J. C.** (2006). American tegumentary leishmaniasis: Is antimonial treatment outcome related to parasite drug susceptibility? *The Journal of Infectious Diseases,* **194**, 1168-1175. doi: 10.1086/507710

14. **Hendrickx, S., Boulet, G., Mondelaers, A., Dujardin, J. C., Rijal, S., Lachaud, L., Cos, P., Delputte, P. and Maes, L.** (2014). Experimental selection of paromomycin and miltefosine resistance in intracellular amastigotes of Leishmania donovani and L. infantum. *Parasitology Research,* **113**, 1875-1881. doi: 10.1007/s00436-014-3835-7.

15. **Inocencio da Luz, R., Romero, G.A., Dorval, M.E., Cruz, I., Cañavate, C., Dujardin, J.C., Van Assche, T., Cos, P., Maes, L.** (2011) Drug susceptibility of Leishmania infantum (syn. Leishmania chagasi) isolates from Brazilian HIV-positive and HIV-negative patients. *Journal of Antimicrobial Chemotherapy*, **66**: 677-679.
